# Researchers should no longer delay implementation of Pap screening in low and middle income countries pending research into novel screening approaches

**DOI:** 10.1186/s13027-024-00576-5

**Published:** 2024-04-26

**Authors:** Eric J Suba

**Affiliations:** 1 Global Cervical Cancer Prevention Project, San Francisco, California USA; 2 National Center for Bioethics in Research and Health Care, Tuskegee University, Tuskegee, Alabama USA

**Keywords:** Cervical cancer screening, Public health policy, Low and middle income countries

## Abstract

A study coordinated by Groesbeck Parham and Mark Schiffman describes a novel approach to single-visit, point-of-care cervical screening and triage for low and middle income countries (LMICs) that uses an HPV screening test that is not affordable in LMICs combined with a triage test that is not available at the point of care. Pap smears are feasible, affordable, and well-suited for single-visit, point-of-care cervical screening and triage in LMICs. Research into a discredited cervical screening test, funded by the US National Cancer Institute, contributed to at least 500,000 preventable cervical cancer deaths by delaying implementation of Pap screening throughout India for 18 years. Researchers should no longer delay implementation of Pap screening in LMICs pending research into novel screening approaches. Instead, researchers should prioritize cervical screening approaches that will save as many lives as quickly as possible in LMICs. To that end, Parham et al. should implement good-quality, single-visit, point-of-care Pap smear screening in LMICs until better-quality, single-visit, point-of-care HPV screening becomes widely affordable in LMICs.

## HPV screening in low and middle income countries (LMICs)

The study coordinated by Groesbeck Parham and Mark Schiffman describes a novel approach to single-visit, point-of-care cervical screening for LMICs that uses HPV typing with the BD Onclarity™ assay system combined with a triage approach assisted by automated visual evaluation (AVE) [1]. Parham et al. acknowledge “the AVE algorithm was not run on a smartphone camera itself (was run offline using high intensive graphic cards/chipsets on computers in the lab). Adapting and miniaturizing high performing AVE algorithms onto lower-cost devices is a major technical and operational requirement for this technology to be transformed into a near-patient/point-of-care clinical application.”

Parham et al. acknowledge “implementing affordable and accurate HPV screening is still a major challenge in lower-resource settings.” For more than 20 years, researchers, including South African physician Lynette Denny [2], have advocated HPV screening for LMICs. In 1999, with an initial gift of US$50 million, the Bill & Melinda Gates Foundation established the Alliance for Cervical Cancer Prevention on the non-transparent central assumption that non-cytologic technologies, rather than Pap screening, were the most likely solutions to the problem of cervical cancer in LMICs [3]. That assumption may have delayed implementation of Pap screening in LMICs. In 2004, with millions more US dollars from the Gates Foundation, Seattle-based PATH partnered with Digene Corporation (subsequently acquired by Qiagen Corporation) to develop HPV tests affordable for LMICs [4]. That initiative failed. In 2023, the US National Cancer Institute (NCI) acknowledged “the WHO Cervical Cancer Elimination strategy calls for screening the majority of women with a high-performance HPV test twice in their lifetime. Realization of that goal using current commercial HPV tests is unlikely.” [5] Parham et al. suggest the Atila Biosystems ScreenFire HPV test may become affordable for LMICs, but do not explain how Atila Biosystems will succeed where the Gates Foundation, PATH, and Digene/Qiagen have failed. Schiffman’s 2009 claims that “affordable and accurate HPV DNA testing is already a reality,” [6] and that “low-resource countries do not need to establish large cytologic-testing (Papanicolaou) programs“[6] appear to have been incorrect, and may have delayed implementation of Pap screening in LMICs.

It would be imprudent to assume that HPV tests which may become affordable for LMICs will be of higher quality than Pap smears. The WHO International Agency for Research on Cancer (WHO/IARC) warns ‘‘increased competition resulting in diminishing market share and reductions in the cost of testing might lead HPV test manufacturers to relax their standards of quality. Such a scenario could prove disastrous in many respects, since there are theoretically many more variables that can affect the performance of HPV testing than there are for Pap screening’’ [7]. In 2022, in response to longstanding concerns regarding HPV test affordability in LMICs, Lynette Denny reflected “there are some days I wake up and I think ‘Come on, let’s forgive the Pap [smear] and let’s go back to cytology [ie Pap smears]’” [8], apparently reflecting, on some days, a preference for Pap screening instead of HPV screening in South Africa.

## Pap screening in LMICs

Pap smears are feasible, affordable, and well-suited for single-visit, point-of-care cervical screening and triage in LMICs. Unlike HPV tests, the Pap smear has features of both a screening test and a diagnostic test. Pap smear results of “high-grade squamous intraepithelial lesion” (HSIL) and “malignant” may be considered diagnostic of high-risk cervical neoplasia (e.g. cervical intraepithelial neoplasia (CIN) grade 2/3 or carcinoma) with no requirement for additional confirmatory testing. Women with lower-grade Pap smear results may be triaged for follow-up care based on results of co-collected Pap smears. Pap smears may be processed either singly or in batches, with turnaround times measurable in minutes between smear collection and final result reporting. Liquid-based cytology makes cytologic screening less affordable but no more accurate [9].

Individuals with positive screening tests for cancer desire to know whether or not they truly have the disease. Women with point-of-care Pap smear results of HSIL or malignant may receive immediate treatment using excisional techniques (e.g. cold knife conization, loop electrosurgical excision procedure, large loop excision of the transformation zone, or laser conization) rather than ablative techniques (e.g. cryotherapy, CO_2_ laser ablation, thermal ablation, diathermy, or cold coagulation) [10]. Unlike ablative treatment techniques, excisional treatment techniques produce tissue samples that may be analyzed to establish or exclude the possibility of invasive carcinoma. Providing ablative treatment to cervical screen-positive women before the possibility of invasive carcinoma has been excluded has problematic implications for provider acceptance, patient safety, and informed consent [11]. In such scenarios, screen-positive women will be informed that they have a positive cervical cancer screening test; that ablative treatment will probably render it impossible for anyone to determine whether cancer is present; and, if cancer is in fact present, that ablative treatment will not be curative [11]. Ablative treatment methods are effective treatment for pre-cancerous cervical lesions, but have not been shown to cure invasive cervical cancers.

In 2005, the Head of Cancer Screening at WHO/IARC emphasized “Our results clearly show that good-quality Pap smear screening can be implemented even in a rural setting of a developing country with reasonable investment, while HPV screening does not give any better detection of CIN2/3 lesions, despite the higher investments” [12]. The HPV test used by WHO/IARC was the Qiagen Hybrid Capture® 2 HPV test, currently priced at US$53 for consumable supplies alone [13]. Pap smears are labor-intensive, with their major cost component being the salary needed to pay a cytotechnologist to analyze each smear (Table 1) [13]. In the USA, where cytotechnologist salaries approximate US$90,000 and pathologist salaries approximate US$500,000, Pap smears are priced at US$15.15, which is affordable for lower-income Americans [13]. Pap smear prices are lower in lower-income settings where, by definition, salaries are lower [13]. LMIC healthcare workers would receive USA-level salaries to implement Pap smear screening for the same investment required to implement HPV screening using HPV tests priced at US$10 each [13].

**Table 1:** Salary, supply, and equipment costs for Pap smears and collection devices (in 2020 US$) [13]

Category	Item	Cost per Pap smear
Salaries	Cytotechnologist screening and interpretation of Pap smear	Cytotechnologist annual salary (in 2020 US$) ÷ 20,000^a^
	Pathologist interpretation of atypical Pap smear	Pathologist annual salary (in 2020 US$) ÷ 120,000^b^
Supplies	Modified wooden Ayre spatula	0.06
	Alcohol fixative	0.06
	Pap smear stains	0.07
	Cover slip	0.06
	25 mm x 75 mm glass slide	0.06
	Mounting medium	0.03
Equipment	Microscope	0.04^c^

^a^assumes a cytotechnologist screens and interprets 20,000 Pap smears per year without any other work responsibilities

^b^assumes 10% of all Pap smears screened by cytotechnologists are atypical and referred to pathologists who spend an average of 10 min interpreting each atypical smear and work 40-hour weeks for 50 weeks each year

^c^assumes a new microscope costs US$8,000 and is used only to screen and interpret 20,000 Pap smears per year for 10 years

Nationwide, more than 5% of women screened through the National Laboratory Network of South Africa present with Pap smear results of HSIL or malignant, with some districts reporting HSIL rates higher than 10% [14]. Nationwide, most women in South Africa with Pap smear results of HSIL or malignant are lost to follow-up [15]. Given the resources that will be required to provide follow-up care to all South African women with Pap smear results of HSIL or malignant, the clinical utility in South Africa of any cervical screening test result other than a Pap smear result of HSIL or malignant is uncertain, as are the benefits of transitioning from Pap smear screening to HPV screening [13]. Published data regarding nationwide Pap smear HSIL rates in other LMICs are less extensive than the data from South Africa.

## Conclusions and recommendations

Opportunity costs, borne by the underserved, are associated with prioritizing research into novel health interventions in settings where established interventions are feasible but unavailable [16]. The US Preventive Services Task Force has determined that Pap screening reduces cervical cancer rates by 60-90% within 3 years of introduction, and that those reductions in suffering and death are “consistent and dramatic​ across populations” [17]. NCI-funded research into a discredited cervical screening test contributed to at least 500,000 preventable cervical cancer deaths by delaying implementation of Pap screening throughout India for 18 years [13, 18]. Researchers should no longer delay implementation of Pap screening in LMICs pending research into novel cervical screening approaches. Instead, researchers should prioritize cervical screening approaches that will save as many lives as quickly as possible in LMICs [13]. To that end [13], Parham et al. should implement good-quality, single-visit, point-of-care Pap smear screening in LMICs until better-quality, single-visit, point-of-care HPV screening becomes widely affordable in LMICs.

## Data Availability

No datasets were generated or analysed during the current study.

## Abbreviations

HPV: human papillomavirus
AVE: automated visual evaluation
WHO: World Health Organization
IARC: International Agency for Research on Cancer
NCI: US National Cancer Institute
LMIC: low and middle income country
HSIL: high-grade squamous intraepithelial lesion
CIN 2/3: cervical intraepithelial neoplasia, grade 2 and/or grade 3
